# Supplementary material for: General Practitioners’, Pharmacists’ and Parents’ Views on Antibiotic Use and Resistance in Malta: An Exploratory Qualitative Study
Source: Antibiotics (Basel). 2022 May 14;11(5):661. doi: 10.3390/antibiotics11050661 (PMC9137633; doi:10.3390/antibiotics11050661)
Supplement: Supplementary file 1 [file antibiotics-11-00661-s001.zip › Suppl material S1_FGD guide (GPs).pdf]

SUPPLEMENTARY MATERIAL S1: FGD GUIDE – GPs

1. **How do you think GPs can help in promoting *judicious* antibiotic consumption (for RTIs)?**
    a. *What I mean by judicious is rational/appropriate consumption*

2. **What challenges do you think GPs face when patients present with respiratory tract complaints in the community?**
    a. What challenges have you personally faced as a GP?
    b. What can be done to alleviate these challenges?
    c. Do you have any suggestions for improvement?

3. **As you probably know, medical reps are a common source of information on antibiotics and resistance for GPs. What are your thoughts on this?**
    a. How does the pharmaceutical industry influence GPs?
    b. What do you think about these influences?

4. **What are your views on the availability of up-to-date National guidelines, based on local microbial and resistance epidemiology, in Malta?**
    a. How important and/or applicable are they for your practice?

5. **Now that we have discussed this issue in greater detail, may you describe some situations in which you would prescribe antibiotics for respiratory tract infections?**
    a. When could one consider this? Why?
    b. According to a Maltese study conducted in 2011, GPs antibiotic prescribing is mostly influenced by the following factors:

Patient characteristics	*Clinical presentation*
Age	Ear infections
Pregnancy/breastfeeding	Sinus infections
Chronic pulmonary disease	Throat infections
Difficult patients	Infections in the bronchi
Patients who return with the same problem	Productive cough with greenish sputum
Socioeconomic factors	Fever higher than 100°F
Occupation	Shortness of breath
	Pustular inflammation of the tonsils
	Signs of otitis media or externa
	Percussion tenderness over the frontal sinuses
	Localized crepitations on chest auscultation
	Enlarged cervical lymph nodes

    c. Agree/disagree? Why/why not?
    d. Are they valid factors?

e. Which other factors might you consider instead?

6. **The latest Eurobarometer data showed that the main reason for prescribed antibiotic use is cold, sore throat and flu.**
    a. Why do you think this is so?
    b. What can be done to improve?
    c. What are your recommendations which could help improve antibiotic prescribing?
