# Supplementary material for: General Practitioners’, Pharmacists’ and Parents’ Views on Antibiotic Use and Resistance in Malta: An Exploratory Qualitative Study
Source: Antibiotics (Basel). 2022 May 14;11(5):661. doi: 10.3390/antibiotics11050661 (PMC9137633; doi:10.3390/antibiotics11050661)
Supplement: Supplementary file 1 [file antibiotics-11-00661-s001.zip › Suppl materials S2_FGD guide (parents).pdf]

SUPPLEMENTARY MATERIAL S2: FGD GUIDE – PARENTS

HEALTH-RELATED DECISION-MAKING

1. **Do you remember the last time you had a sore throat, runny nose, chesty cough and/or fever?**
    - What did you do when you experienced these symptoms?
    - Why did you do that?
    - How would you have felt if you did not meet a medical professional/get treatment?

2. **If your own children had these symptoms, how would you act?**
    - Would you act differently? If yes, how so?

GENERAL KNOWLEDGE

3. **Can you tell me what you know about antibiotics?**
    - What can they be used for?
    - What should they <u>not</u> be used for?

4. **What do you think causes antibiotic resistance?**
    - How problematic do you think antibiotic resistance is in Malta/the rest of the world?

PATIENT-DOCTOR INTERACTION

When you visit your doctor for a <u>respiratory tract problem</u> (give examples if necessary)…

5. **What do you expect to get out of the visit?**
    - What kind of medications?
    - What kind of advice?

6. **Sometimes patients specifically ask their doctor for antibiotics. What do you think about this?**
    - Have you ever done this before? May you explain the situation further?

CLIENT-PHARMACIST RELATIONSHIP

When you go to a pharmacist for a <u>respiratory tract problem</u> (give examples if necessary)…

7. **How often do you visit a pharmacist without visiting a doctor first, if at all?**

8. **What do you expect from the pharmacist when you have a respiratory tract problem?**
    - What kind of medications?
    - What kind of advice?

9. **Sometimes clients specifically ask pharmacists for antibiotics. What do you think about this?**

- Have you ever done this before? May you explain the situation further?
- Would you do this even if the doctor did not prescribe antibiotics?

ANTIBIOTIC USE, STORAGE AND DISPOSAL

10. Sometimes people take antibiotics without consulting a doctor first. Have you ever done this or thought of doing this?
- How do you get a hold of the antibiotics?

11. How likely are you to follow your doctor's/pharmacist's instructions on how to take the antibiotics prescribed?
- Why would you do that?

12. Doctors sometimes choose to delay antibiotic prescriptions. What I mean is that they may either ask you to wait a couple of days and come back if symptoms get worse, or they might give you a prescription but instruct you not to buy it for a couple of days if symptoms get worse. What are your thoughts on this practice?
- What would you do with the delayed prescription?

13. Sometimes when you finish an antibiotic course you may end up with leftovers. What do you do with leftover antibiotics?
- How do you use the leftovers?
- How do you dispose of the leftovers?

ROLE IN TACKLING ANTIBIOTIC RESISTANCE

14. Whose responsibility is it to help combat this problem?

15. How do you think we can combat this problem?
- What can you do?
