# Supplementary material for: General Practitioners’, Pharmacists’ and Parents’ Views on Antibiotic Use and Resistance in Malta: An Exploratory Qualitative Study
Source: Antibiotics (Basel). 2022 May 14;11(5):661. doi: 10.3390/antibiotics11050661 (PMC9137633; doi:10.3390/antibiotics11050661)
Supplement: Supplementary file 1 [file antibiotics-11-00661-s001.zip › Suppl materials S3_FGD guide (pharmacists).pdf]

SUPPLEMENTARY MATERIAL S3: FGD GUIDE – PHARMACISTS

AWARENESS AND UNDERSTANDING ON ANTIBIOTIC MISUSE AND ANTIBIOTIC RESISTANCE

1. **How do you look upon antibiotic use and resistance in Malta?**
    - What factors do you think are contributing to resistance in Malta?
    - How, in your opinion, are antibiotics misused in Malta?

2. **How do you perceive the general population's awareness on antibiotic use and resistance?**

3. **What are your opinions on the prescribing practices of local GPs?**

ANTIBIOTIC DISPENSING BEHAVIOUR

4. **When/under what circumstances do you dispense antibiotics?**

5. **At times antibiotics are dispensed without a prescription. Can you explain under what circumstances this could happen?**
    - What factors influence your decision to dispense an antibiotic without a prescription?
    - How would you choose what antibiotic to dispense?

6. **How often are antibiotic prescriptions retained?**
    - When would you give the prescription back to the client?

7. **What would you likely do if a client comes with an old/expired antibiotic prescription?**

8. **Sometimes clients could insist that you dispense antibiotics to them. Has this ever happened to you?**
    - How would you act in such situations?

9. **What do you understand by 'delayed antibiotic prescription'?**
    - What are your opinions on this?
    - How often do clients ask for the antibiotic despite it being delayed by the doctor?
    - How do you act in such circumstances?

PHARMACIST-GP INTERACTION

10. **How would you describe your relationship with GPs?**
    - How often do you interact?
    - How do you communicate about their prescriptions?

11. How do you feel about pointing out inappropriate prescriptions/prescription errors to GPs?
- What else could you do?

EDUCATIONAL INITIATIVES

12. How do you look upon your role as educator?

PHARMACIST'S ROLE IN TACKLING ANTIBIOTIC RESISTANCE

13. Whose responsibility is it to help combat antibiotic misuse in the community and antibiotic resistance?
- What can you do?
    - As pharmacists
    - As members of the community

SUGGESTIONS FOR SUITABLE INTERVENTIONS

14. What do you think needs to be done in Malta to tackle antibiotic misuse?
- To improve the situation?
- To strengthen what is already working well?
